# MANTA vs Perclose for Large-Bore Femoral Closure in Impella-Assisted High-Risk Percutaneous Coronary Intervention: Retrospective Outcomes Study

**DOI:** 10.1016/j.jscai.2025.103943

**Published:** 2025-10-30

**Authors:** Muhammad Memon, Raef Ali Fadel, Herbert D. Aronow, Ahmad Jabri, Mohammad Alqarqaz, Khaldoon Alaswad, Asaad Nakhle, Mir Babar Basir, Pedro Villablanca

**Affiliations:** a Department of Internal Medicine, Henry Ford Health System, Detroit, Michigan; b Division of Cardiology, Henry Ford Health System, Detroit, Michigan; c Interventional Cardiology, Henry Ford Health System, Detroit, Michigan; d Department of Structural Heart Disease, Division of Cardiology, Henry Ford Health System, Detroit, Michigan

**Keywords:** femoral access, high-risk PCI, Impella, large-bore, MANTA, Perclose, vascular closure device

## Background

Temporary mechanical circulatory support (tMCS) is frequently used in complex high-risk (HR) percutaneous coronary intervention (PCI), particularly in patients with severe ventricular dysfunction or extensive coronary disease. While the Impella percutaneous left ventricular assist device provides effective hemodynamic support during these procedures,^1^ device removal and closure of the large-bore arterial access site remain a key challenge, primarily due to risk of bleeding and other vascular access site complications.^2^ Vascular closure devices (VCDs) can facilitate achievement of hemostasis in this setting, with the most common being the suture-based Perclose^3^ and collagen plug–based MANTA closure devices.^4^ Despite widespread use, comparative data examining efficacy and vascular complications, especially in the context of Impella-assisted HR-PCI, remains limited.^1^ Device selection is often based on operator preference, vascular anatomy, and other patient-specific factors.^5^ We conducted a retrospective analysis at a tertiary high-volume center to evaluate VCD outcomes associated with MANTA vs Perclose in patients undergoing Impella-assisted HR-PCI.

## Materials and methods

Our study included patients who underwent Impella-assisted HR-PCI from 2018-2023 at our center and received either a MANTA or a Perclose VCD as index closure device for hemostasis. Device choice was at operator discretion but informed by femoral angiography and ultrasound. No cases had dedicated computed tomography imaging before PCI. MANTA depth was consistently reported in every case of upfront MANTA use; all MANTA devices were of 14F. Only elective or staged HR-PCI cases were included. Patients presenting in cardiogenic shock were excluded. Most cases involving Perclose (82%) were preclosed with deployment of the Perclose device before insertion of a 14F Impella sheath. All Perclose cases involved 2-device preclosure. The Impella was removed at the end of the procedure in 78% of cases receiving MANTA closure and 80% of cases receiving Perclose. Patients were excluded if they died before Impella removal. If closure was delayed, a new MANTA device was used. Outcomes included, vascular complications requiring intervention, Bleeding Academic Research Consortium type 3 and type 5 major bleeding (defined by Bleeding Academic Research Consortium criteria), and successful closure (defined as hemostasis without need for further intervention or major bleeding).

## Results

**Figure 1:**
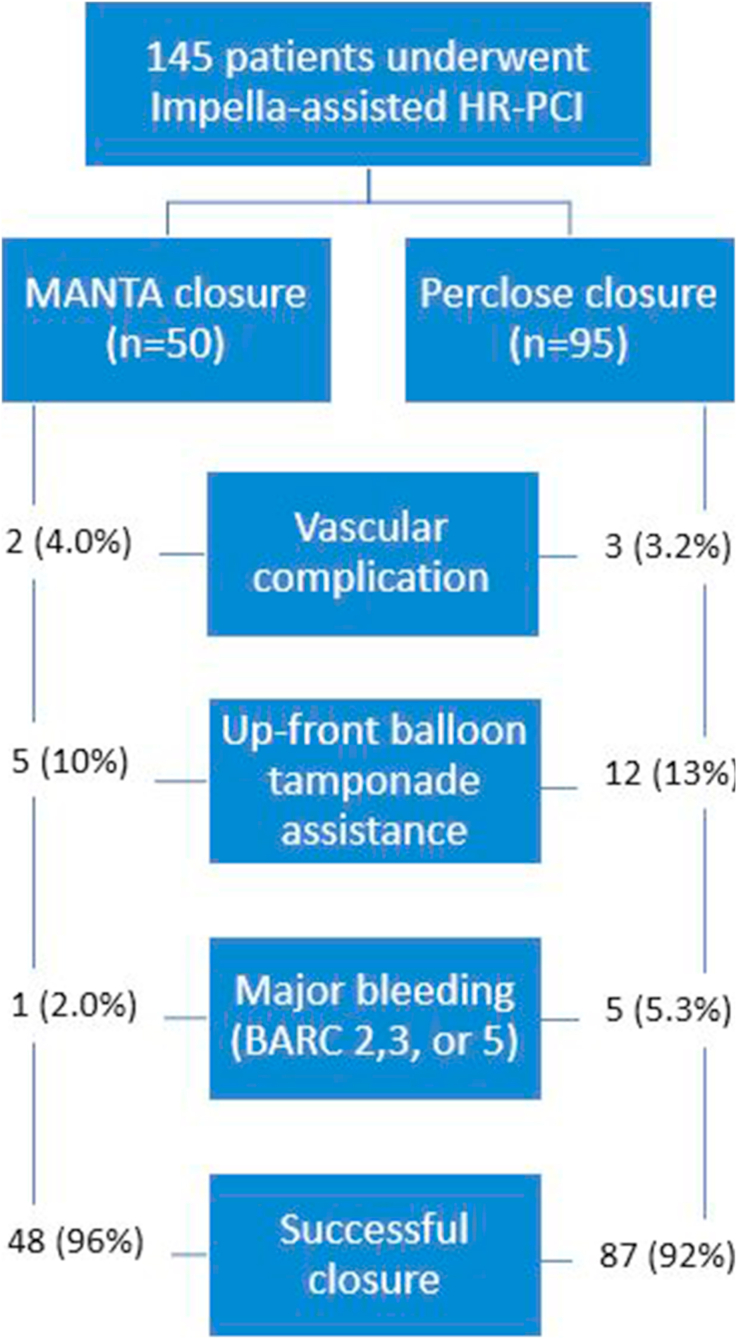
**Comparative outcomes after Impella removal using MANTA vs Perclose.** Blue boxes display rates of closure success, major bleeding, vascular complications requiring intervention, and use of up-front balloon tamponade. Sample sizes: MANTA n = 50, Perclose n = 95. Observed event rates were: closure success 96% vs 92%, major bleeding 2.0% vs 5.3%; vascular complications 4.0% vs 3.2%, balloon tamponade 10% vs 13%. All patients had femoral Impella access. Major bleeding was defined by Bleeding Academic Research Consortium (BARC) criteria; “successful closure” was defined as hemostasis without need for further intervention or major bleeding. Values are n (%). HR-PCI, high-risk percutaneous coronary intervention.

A total of 145 patients were analyzed: 50 received MANTA (34.5%) and 95 received Perclose (65.5%). Patients in the Perclose group were older (75.8 vs 71.1 years; *P* = .009), but other baseline factors such as peripheral artery disease (25.2%) and SYNTAX scores (∼29) were similar. All patients had femoral Impella access, and none required escalation of tMCS intraprocedurally or postprocedurally. The Impella was removed at the end of the procedure in 78% of cases receiving MANTA closure and 80% of cases receiving Perclose. For cases receiving Perclose, 82% were deployed before implantation of the Impella (ie, preclose). All cases receiving MANTA had reported MANTA depth before deployment for accurate assessment of arteriotomy depth, and all MANTA devices used in the study were of 14F. Upfront balloon tamponade was used in 10% of MANTA cases and 13% of Perclose cases, with the decision based on operator preference, vessel anatomy assessment, access site complexity, and patient bleeding risk factors. Of the 8 Perclose closure failures (8%), 3 required balloon tamponade with covered stenting, and 5 required balloon tamponade with manual compression, with 1 case receiving Angio-Seal. Of the 2 MANTA failures (4%), 1 required balloon tamponade with covered stenting and 1 required balloon tamponade with manual compression. Vascular complications occurred in 2 (4.0%) of MANTA cases (both hematomas) and 3 (3.2%) of Perclose cases (including 2 hematomas and 1 limb ischemia). Major bleeding occurred in 2.0% of MANTA cases and 5.3% of Perclose cases. Closure success was high in both groups: 96% for MANTA and 92% for Perclose (Figure 1).

## Discussion

In this real-world experience, both MANTA and Perclose demonstrated high procedural success and low complication rates in the hands of experienced operators. Although conducted at a single center, this represents one of the largest cohorts to date exclusively examining vascular closure outcomes in Impella-supported HR-PCI, specifically comparing MANTA and Perclose devices. These findings contribute to the growing evidence base regarding optimal patient selection for each closure method and provide important safety and efficacy data for both devices. Future multicenter studies will be essential to validate these results and further refine patient selection criteria for vascular closure in this HR population. The procedural consistency and relatively large cohort add to the relevance of the findings. The fact that none of the patients required tMCS escalation supports that closure outcomes were not confounded by unstable clinical scenarios. It is important to note that our institutional practice of routinely leaving a safety wire in place during MANTA vascular closure device deployment facilitates rapid balloon tamponade in the event of access site complications and may contribute to the low vascular complication rates observed in our series.^6^

Current literature is somewhat mixed regarding vascular closure device performance. Randomized controlled trials (RCTs) consistently demonstrate that ProGlide is associated with fewer access site related vascular complications compared with MANTA. Meta-analyses evaluating RCTs show that MANTA carries a higher risk of access site complications (relative risk, 1.70; 95% CI, 1.16-2.51) and increased need for endovascular stenting or vascular surgery due to device failure (RR, 3.53; 95% CI, 1.07-11.33) compared to ProGlide.^7^ Individual RCTs consistently fail to demonstrate MANTA superiority and often report numerically higher complication rates.

Conversely, retrospective and observational studies frequently report more favorable or equivalent outcomes for MANTA, including lower rates of vascular and bleeding complications, shorter hospital stays, and reduced need for additional closure devices.^8^

While closure methods were not standardized in PROTECT II or III, bleeding was a key contributor to adverse events.^9^^,^^10^ MANTA’s US pivotal trial reported rapid hemostasis (∼24 seconds) with complication rates similar to historical suture-based data, supporting its use for 10F to 20F access.^6^ In transcatheter aortic valve replacement populations, results have been mixed: 1 study showed fewer complications with MANTA (10.7% vs 19.0%),^7^ while another randomized trial reported higher minor complications with MANTA.^11^ A 2023 meta-analysis found no significant differences in bleeding or mortality between MANTA and suture devices.^1^ In Impella-specific studies, 1 found significantly lower bleeding with MANTA than that with manual compression (6.5% vs 37.9%),^10^ while others have demonstrated high success with preplaced Perclose.^3^

The choice between these devices often depends on technical experience. The MANTA is quicker to deploy but requires a straight vessel and minimum diameter (∼6.0 mm).^5^ Perclose allows multiple attempts and more flexibility if the initial closure attempt fails. Our data support that either VCD can be used effectively, particularly when matched to patient anatomy and operator familiarity. The slightly higher use of balloon tamponade and bleeding in the Perclose group may reflect greater caution or procedural complexity in an older cohort, although this requires further exploration.

### Limitations

This study has limitations. It was retrospective, nonrandomized, and conducted at a single high-volume center. Device selection was not randomized and likely influenced by anatomical and procedural factors. Specific information on calcification that may have impacted selection was not collected, as much of these data were gathered at the time of procedure via ultrasound, which were not recorded in the medical chart. While baseline characteristics were well balanced, residual confounding was possible. The sample size may not detect smaller differences between groups. Additionally, while we elected not to perform formal analysis by operator, it is important to note that individual operators demonstrated clear device preferences throughout the study period, introducing a potential confounding variable related to operator experience that could have influenced our comparative effectiveness findings.

## Conclusion

In conclusion, both MANTA and Perclose offer safe and effective large-bore femoral closure in patients undergoing Impella-supported HR-PCI. As bleeding remains a key modifiable complication, optimizing closure strategy is essential. Larger prospective studies are needed to establish evidence-based protocols that account for anatomy, procedural context, and operator proficiency. Future research should explore hybrid closure techniques and newer closure devices. Until such data are available, individualized, anatomy-based decision making remains the best approach.
